# Validation of a same-day real-time PCR method for screening of meat and carcass swabs for *Salmonella*

**DOI:** 10.1186/1471-2180-9-85

**Published:** 2009-05-07

**Authors:** Charlotta Löfström, Michael Krause, Mathilde H Josefsen, Flemming Hansen, Jeffrey Hoorfar

**Affiliations:** 1 National Food Institute, Technical University of Denmark, Mørkhøj Bygade 19, 2860 Søborg, Denmark; 2 Danish Meat Research Institute, Maglegårdsvej 2, 4000 Roskilde, Denmark

## Abstract

**Background:**

One of the major sources of human *Salmonella *infections is meat. Therefore, efficient and rapid monitoring of *Salmonella *in the meat production chain is necessary. Validation of alternative methods is needed to prove that the performance is equal to established methods. Very few of the published PCR methods for *Salmonella *have been validated in collaborative studies. This study describes a validation including comparative and collaborative trials, based on the recommendations from the Nordic organization for validation of alternative microbiological methods (NordVal) of a same-day, non-commercial real-time PCR method for detection of *Salmonella *in meat and carcass swabs.

**Results:**

The comparative trial was performed against a reference method (NMKL-71:5, 1999) using artificially and naturally contaminated samples (60 minced veal and pork meat samples, 60 poultry neck-skins, and 120 pig carcass swabs). The relative accuracy was 99%, relative detection level 100%, relative sensitivity 103% and relative specificity 100%. The collaborative trial included six laboratories testing minced meat, poultry neck-skins, and carcass swabs as un-inoculated samples and samples artificially contaminated with 1–10 CFU/25 g, and 10–100 CFU/25 g. Valid results were obtained from five of the laboratories and used for the statistical analysis. Apart from one of the non-inoculated samples being false positive with PCR for one of the laboratories, no false positive or false negative results were reported. Partly based on results obtained in this study, the method has obtained NordVal approval for analysis of *Salmonella *in meat and carcass swabs. The PCR method was transferred to a production laboratory and the performance was compared with the BAX *Salmonella *test on 39 pork samples artificially contaminated with *Salmonella*. There was no significant difference in the results obtained by the two methods.

**Conclusion:**

The real-time PCR method for detection of *Salmonella *in meat and carcass swabs was validated in comparative and collaborative trials according to NordVal recommendations. The PCR method was found to perform well. The test is currently being implemented for screening of several hundred thousand samples per year at a number of major Danish slaughterhouses to shorten the post-slaughter storage time and facilitate the swift export of fresh meat.

## Background

One of the major sources of human *Salmonella *infection is meat, including pork and poultry [1,2] and therefore efficient and rapid monitoring of *Salmonella *in the meat production chain is necessary. Traditional bacteriological detection of *Salmonella *in foods and environmental samples is costly, laborious, and time-consuming, requiring 3–7 days to obtain a confirmed result [3]. Thus, rapid and cost-effective detection of *Salmonella *is of major interest to the food industry and the public. Real-time PCR technology offers several advantages compared with classical bacteriology in terms of speed, detection limit, potential for automation, and cost [4]. However, it is essential that new PCR methods are reliable, robust and comply with the legislative demand of detecting as few as one *Salmonella *bacterium per 25-g sample. Furthermore, they should be validated against reference culture methods, and last, but not least, be sufficiently robust to be transferred from the expert laboratory to end users.

There are several real-time PCR methods available for the detection of *Salmonella *in various kinds of food [5,6] and carcass swabs [7]. Furthermore, a number of commercial real-time PCR systems have been validated for testing of *Salmonella *in meat and swab samples [5,8-10]. Some of these systems detect *Salmonella *as fast as 9–10 h in meat samples (iQ Check Salmonella II, Bio-Rad, Hercules, CA and GeneDisc, GeneSystems, Bruz, France), but the total time for analysis of carcass swab samples is 17–20 h. Recently, a non-commercial real-time PCR method for detection of *Salmonella *in milk powder [11] has been validated in a multicenter trial. However, to our knowledge, there are no reports on multicenter validation trials where non-commercial methods are evaluated for the detection of *Salmonella *in meat or carcass swabs using real-time PCR.

The objective of this study was to validate a previously developed real-time PCR method [6,12,13] for use as a routine and on-site analysis method for the meat industry. The validation study was performed according to the protocol recommended by the validation body of the Nordic countries (NordVal) [14,15], including comparative and collaborative trials on minced pork and veal meat, chicken neck-skins and pig carcass swab samples. The method is based on a shortened (compared to the NMKL-71 method) pre-enrichment in buffered peptone water (BPW) followed by automated DNA purification and subsequent detection using real-time PCR. In this method, a part of the *ttr*RSBCA locus specific for *Salmonella *is amplified giving a high selectivity [6]. The PCR method used includes an internal amplification control (IAC), making it useful as a diagnostic tool. The overall time for the analysis of meat samples is 14 h, and for carcass swab samples 16 h. Both time-spans are operational for two-shift work at slaughterhouses. The method has on the basis of results obtained in this study together with already published data on selectivity [6] gained NordVal approval and is currently being implemented at major Danish meat producers.

## Results

### Comparative trial

The comparative trial was conducted in accordance with the guidelines provided by NordVal [15] and included the matrices meat (minced pork and veal meat as well as poultry neck-skins) and environmental samples (swabs from pig carcasses). The relative detection level, accuracy, sensitivity and specificity were evaluated for the real-time PCR method in comparison with the reference culture-based method currently in use (Table 1)[3].

**Table 1:** Results obtained in the comparative trial by the real-time PCR and the reference culture method ^a, b^.

Sample type^c^	No. of samples						% Value^d^			κ^e^
			
	N	PA	NA	FN	TP	FP	AC	SE	SP	
Minced meat	60	30	30	0	0	0	100	100	100	1.00
Poultry neck-skins	60	27	31	0	2	0	97	107	100	0.97
Pig carcass swabs	120	21	98	1	0	0	99	95	100	0.97

**TOTAL**	**240**	**78**	**159**	**1**	**2**	**0**	**99**	**103**	**100**	**0.97**

^a ^PA: Positive Agreement, NA: Negative Agreement, TP: True Positive, FN: False Negative, FP: False Positive, AC: Relative Accuracy, SE: Relative Sensitivity, SP: Relative Specificity, N = PA +NA + FN + TP + FP.

^b ^Results are given after confirmation.

^c ^Matrices as defined by NordVal [15]; matrix meat: minced meat (raw pork and veal) and poultry neck skins, matrix environmental samples: pig carcass swabs. Meat samples were artificially contaminated and swab samples potentially naturally contaminated.

^d ^See Materials and Methods for accuracy, sensitivity and specificity equations.

^e ^Cohen's kappa calculated according to NMKL procedure no. 20 [26].

The detection level of the two methods was 1–10 CFU/25 g sample (corresponding to a relative detection level of 100%) in all cases except for the swabs inoculated with *S*. Enteritidis, where it was 10–100 CFU/25 g for the NMKL method (relative detection level > 100%) (data not shown). To determine the relative accuracy, sensitivity and specificity, a total of 240 samples representing meat and environmental samples were analyzed by the PCR and NMKL methods (Table 1). A total of 80 out of 240 samples gave positive results by real-time PCR, compared with a total of 79 by the culture-based method. Two samples showed positive deviation (true positives by the PCR method) and one negative deviation (false negative by the PCR method) (Table 1). A very good agreement between the two methods was obtained using Cohen's kappa (Table 1).

### Collaborative trial

The purpose of the collaborative trial was to determine the variability in the results obtained by the real-time PCR method detecting *Salmonella *in identical samples. The trial was conducted in accordance with the guidelines provided by NordVal [15]. The samples and the other contents of the ring trial kit sent out to the participants were found to be stable during the period of the trial (data not shown). The influence of the refrigerated transit was investigated prior to the collaborative trial, and no detrimental effects were found after three days (data not shown).

Six laboratories participated in the collaborative trial, and valid results were obtained from five of the laboratories and used for the statistical analysis (Table 2). In agreement with the predefined criteria, results from one participant were excluded due to failure in the PCR analysis (lack of amplification in the positive control and several samples with no amplification of either the target or the IAC). The unexpected PCR results obtained by this participant were probably caused by a delay in the transport of the ring trial package (> 5 days). Statistical analysis of the results from the remaining five laboratories gave a relative specificity, sensitivity and accuracy of 100% for all of the tested matrices at all three inoculation levels, except for the relative accuracy for swab samples which was 83% when all inoculation levels were analyzed together. For the positive control samples containing *Salmonella *DNA, a Ct value of 32.6 ± 1.6 was obtained for the five laboratories. There were small variations in the Ct values obtained for duplicate samples of the same matrix at the same spiking level analyzed at each laboratory (standard deviation 0.0–2.7) as well as for the same sample analyzed by each laboratory (standard deviation 1.1–1.9).

**Table 2:** Collaborative trial: PCR results for *Salmonella *in artificially contaminated meat samples and pig carcass swabs.

Sample type	Participant no.	Ct values for replicates from indicated level of spiking (CFU/25 g)^a^		
		
		0	1–10	10–100
Carcass swabs	1	> 36, > 36	17, 19	19, 19
	2	> 36, > 36	14, 16	16, 16
	3	> 36, > 36	15, 17	16, 16
	4	> 36, > 36	16, 18	17, 17
	5	> 36, 34	16, 18	19, 17
	Mean ± SD^b^	n.a.^c^	16.5 ± 1.3	17.1 ± 1.3
				
Poultry neck-skins	1	> 36, > 36	28, 28	25, 24
	2	> 36, > 36	26, 26	24, 24
	3	> 36, > 36	29, 28	25, 24
	4	> 36, > 36	24, 25	23, 22
	5	> 36, > 36	25, 25	22, 23
	Mean ± SD^b^	n.a.	26.6 ± 1.8	23.6 ± 1.1
				
Minced meat	1	> 36, > 36	20, 21	17, 17
	2	> 36, > 36	21, 20	16, 18
	3	> 36, > 36	19, 19	16, 15
	4	> 36, > 36	18, 18	13, 14
	5	> 36, > 36	18, 18	17, 13
	Mean ± SD^b^	n.a.	19.4 ± 1.9	15.4 ± 1.8

^a ^Ct values below 36 were considered as positive responses.

^b ^The mean and standard deviation calculated for all the replicate analysis of the same sample independent of the participant.

^c ^n.a.: not applicable

### External validation

In order to evaluate the performance of the real-time PCR method on-site, it was transferred and implemented at a production laboratory previously using PCR-based analysis with the BAX system. Artificially contaminated pork filet samples (n = 39) were analyzed in parallel with the real-time PCR and BAX methods. In general, a good agreement (κ = 0.77) was found between the two methods based on the results from the 39 artificially contaminated samples (Tables 3 &4). The real-time PCR method detected 33 of the 39 samples inoculated with *Salmonella*, whereas the BAX system detected 34 of the 39 samples.

**Table 3:** Results obtained by the real-time PCR and the *Salmonella *BAX PCR in the external validation.

*Salmonella *level (CFU/25-g sample)	No. of samples analyzed	**Result obtained by the PCR and BAX methods**^a^				
		
		PA	PD	ND	NA	Inconc./+
1000	3	3	0	0	0	0
100	3	3	0	0	0	0
10	9	7	0	0	2	0
5	12	10	1	0	0	1
2	12	9	0	1	2	0

**TOTAL**	**39**	**32**	**1**	**1**	**4**	**1**

^a ^PA: positive by PCR and BAX methods, PD: positive by PCR and negative by BAX, ND: negative by PCR and positive by BAX, NA: negative by PCR and BAX methods, inconc./+: inconclusive result by PCR (need re-analysis) and positive by BAX.

**Table 4:** Detailed results from the external validation study.

Salmonella serotype	Inoculation level (cfu/25 g)	**Real-time PCR**^a^			Salmonella BAX Detection System
			
		Ct-value for Salmonella	Ct-value for IAC	Final result	Final result
Infantis	1000	20.05	27.89	Positive	Positive
	100	21.66	29.09	Positive	Positive
	10	27.14	28.68	Positive	Positive
	10	30.59	28.95	Positive	Positive
	10	24.92	28.89	Positive	Positive
	5	29.42	29.09	Positive	Positive
	5	26.57	28.81	Positive	Positive
	5	26.29	27.66	Positive	Positive
	5	26.63	28.79	Positive	Positive
	2	27.70	28.42	Positive	Positive
	2	25.68	28.08	Positive	Positive
	2	27.86	28.56	Positive	Positive
	2	27.20	28.90	Positive	Positive

Agona	1000	22.47	28.97	Positive	Positive
	100	24.70	27.93	Positive	Positive
	**10**	**> 36**	**29.21**	**Negative**	**Negative**
	**10**	**> 36**	**29.07**	**Negative**	**Negative**
	10	26.04	28.93	Positive	Positive
	5	28.47	28.76	Positive	Positive
	**5**	**32.93**	**28.53**	**Positive**	**Negative**
	5	29.84	28.92	Positive	Positive
	5	32.17	27.90	Positive	Positive
	**2**	**> 36**	**28.76**	**Negative**	**Positive**
	**2**	**> 36**	**29.07**	**Negative**	**Negative**
	2	33.22	28.77	Positive	Positive
	2	30.61	27.96	Positive	Positive

Infantis	1000	19.59	29.01	Positive	Positive
	100	23.74	28.86	Positive	Positive
	10	25.55	28.45	Positive	Positive
	10	24.85	28.40	Positive	Positive
	10	26.82	28.36	Positive	Positive
	5	29.82	29.10	Positive	Positive
	5	29.03	28.16	Positive	Positive
	5	24.77	28.28	Positive	Positive
	**5**	**> 36**	**> 40**	**Inconclusive**	**Positive**
	2	28.61	27.88	Positive	Positive
	2	26.24	28.79	Positive	Positive
	2	26.02	28.82	Positive	Positive
	**2**	**> 36**	**28.63**	**Negative**	**Negative**

Results from 39 pork meat samples inoculated with salmonella at different levels and analyzed in parallel on-site using the real-time PCR and the Salmonella BAX methods.

^a ^Samples with a Ct value > 36 is considered negative if the Ct value for the IAC is < 40 and inconclusive if a Ct > 40 is obtained for the IAC. According to the Method Directive for the PCR method, re-analysis of the extracted DNA by PCR is then needed.

## Discussion

The real-time PCR method validated in the present study is intended as a diagnostic tool for routine use in the meat industry, and therefore has specific demands on speed, ease of automation as well as robustness and reproducibility. Furthermore, the method must be specific for *Salmonella *and have detection limit comparable with or better than the culture-based methods in use today as official methods. Using the PCR method, the total time for the analysis of *Salmonella *in meat samples was decreased from at least 3 days for the standard culture-based method [3] to 14 h for meat samples and 16 h for swabs. The time for analysis is comparable with the fastest validated DNA-based analysis kit (e.g. from Bio-Rad and GeneSystems) on the market for meat samples and 1–3 h shorter for swab samples. For the meat producer, this means that the meat can be released faster, leading to decreased costs for storage and prolonged shelf life at the retailers. Implementing this method would allow faster release of *Salmonella*-free fresh meat and meat products.

The sample preparation in the PCR method consists of non-selective enrichment in BPW followed by centrifugation and automated DNA extraction. The use of automated DNA extraction in combination with the closed system of real-time PCR provides a fast and less laborious method with minimized risk of contamination. Furthermore, the real-time PCR method can easily be adapted to include the dUTP-uracil-*N*-glycosylase (UNG) system, minimizing the risk of carryover contamination [16]. The PCR reagents used in the method can be mixed in advance, distributed in smaller, ready-to-use quantities, and frozen at -20°C for up to 3 months [17]. These features are a major benefit for on-site use of the test at the slaughterhouses. The method is an open-formula technique, i.e., the reagents and target gene, etc., are known, in contrast to commercial kits. However, further decreasing the total time for analysis to below 8 h will certainly be even more beneficial to industry and is a challenge in the further developing of the method.

The prevalence of *Salmonella *in Danish pork meat and broiler flocks is low (0.9% and 2.2%, respectively [18]). Therefore, samples artificially contaminated with *Salmonella *in the exponential growth phase stressed by a cold storage overnight to simulate the condition under production of poultry and pork meat were used for the majority of the samples included in the validation study. This alternative to naturally contaminated samples is in compliance with international guidelines [15,19]. However, naturally contaminated swab samples were used for the comparative trial. The NMKL-71 (1999) method [3] was chosen as the reference method because it is used in the Nordic countries instead of the ISO 6579:2002 method [20]. The difference in the two methods is that in the NMKL method only one selective enrichment media is used Rappaport Vassiliades soy broth (RVS) instead of two in the ISO method (RVS and Muller-Kauffmann Tetrathionate-Novobiocin broth, MKTTn). The methods have been determined to be equal to the respective part of the ISO method [21].

The real-time PCR method amplifies a part of the *ttrRSBCA *locus used for tetrathionate respiration in *Salmonella*. The relative selectivity of the PCR assay (primers and probes) has previously been found to be 100% when tested on 110 *Salmonella *strains and 87 non-*Salmonella *strains [6]. Therefore, this parameter was excluded from the comparative test performed in this study, in accordance with NordVal guidelines. The relative accuracy, sensitivity and specificity were evaluated for the PCR method in comparison with the standard culture-based method currently in use for detection of *Salmonella *[3] according to the NordVal protocol (Table 1). Two of the artificially contaminated poultry neck-skins were found positive by the real-time PCR method and negative by the reference method. These samples were considered as true positives because according to ISO 20838:2006 [22] no further verification of positive samples is necessary, as the real time PCR analysis contains a DNA probe specific for the target *Salmonella *gene (*ttrRSBCA *locus). The relative sensitivity for the matrices meat and environmental samples, as well as when all the samples were analyzed together were above 95%, which is the limit considered acceptable according to NordVal [15]. No recommendations concerning the levels for the relative accuracy and relative specificity are given in either the guideline [15] or in the ISO16140 standard [19].

In the collaborative study, complete agreement between the real-time PCR method and the culture-based reference method was obtained for all test characteristics for minced pork and veal meat as well as for poultry neck-skin samples. For carcass swabs, one of the samples that were not artificially contaminated was positive when analyzed by one of the laboratories. However, investigations after the finalization of the trial pointed to a mix-up of two samples during the set-up of the PCR plate, which presents a reasonable explanation for this false-positive result. One of the participants was excluded from the study, due to too long transportation time (> 5 days) which has a detrimental effect on the PCR master mix. There are some limitations to this study that should be taken into consideration when implementing the method at other laboratories. Firstly, only one brand of PCR thermo cycler was used in the study. It has previously been reported that PCR results might vary considerable between different thermocyclers [12] and it might be necessary to adjust reagent concentrations and the temperature program slightly to optimize the method. Secondly, the enrichment step of the method was only performed at the expert laboratory and pellets were sent out for DNA extraction and PCR analysis. Thus the reproducibility was assessed for the DNA extraction and PCR steps. This procedure was approved in advance by NordVal. The participating laboratories were experienced laboratories that were familiar with culture based methodologies. However, in other guidelines for collaborative studies, such as ISO 16140, it is recommended that the complete procedure is performed by all participating laboratories [19].

In the last part of the study, the robustness of the method was verified externally for artificially contaminated pork samples. No significant difference in the result for the real-time PCR method and a commercial SYBR-Green PCR-based analysis system (BAX) was found. However, results were available after 14 h for the real-time PCR method, compared with 20–24 h for the BAX system. In this study, two samples inoculated with a very low level (estimated 2 CFU/25 g) and two samples inoculated at 10 CFU/25 g were negative in both methods, most likely indicating that no surviving *Salmonella *actually were present in the sample. Freezing at -18°C will kill some of the inoculated *Salmonella *cells, thereby affecting the possibility for further detection using BAX or the real-time PCR method. Furthermore, the BAX system failed to detect one sample inoculated with 5 CFU/25 g of *S*. Agona. The same sample was detected using the real-time PCR method although the Ct value was rather high (Ct value of 33). Finally, two samples (5 CFU/25 g of *S*. Infantis and 2 CFU/25 g of *S*. Agona) were not detected by the real-time PCR method although being positive with the BAX system. For one of these samples, however, the IAC was negative as well, prompting a re-examination of the sample. However, at low inoculation levels the cell number added can vary due of statistical reasons thereby affecting the probability of detection [23]. From these data, it can be concluded that the real-time PCR is equivalent to the BAX system in detecting *Salmonella *in artificially contaminated meat samples

## Conclusion

In conclusion, the real-time PCR method was validated in comparative and collaborative trials according to guidelines given by NordVal. The PCR method was found to perform well. Results from this study together with published data on selectivity of the real-time PCR assay [6] formed the basis for obtaining NordVal approval as an alternative method for detection of *Salmonella *in meat and environmental (carcass swabs) samples [24]. After a successful comparison with a commercially available SYBR-Green PCR-based method currently used by a number of meat producers, the real-time PCR method is now being implemented as a routine analysis method by leading poultry and pork producers in Denmark for qualitative detection of *Salmonella *in raw meat and carcass swabs.

## Methods

### DNA extraction

Five-ml aliquots from the pre-enrichments were drawn for DNA-extraction. For the automated DNA extraction method, the aliquots were centrifuged at 3000 × g for 5 min, and DNA-extraction performed on a KingFisher (Thermo Labsystems, Helsinki, Finland), as previously described [13], using a DNA isolation kit for blood, stool, cells and tissue (Magnesil KF, Genomic system, Promega, Madison, WI) as specified by the manufacturer with a total of 75 μl of magnetic particles.

### Real-time PCR

A TaqMan real-time PCR method [6], targeting a region within the *ttrRSBCA *locus, for the specific detection of *Salmonella*, was employed as previously described [13] using 9 μl of the purified DNA as template in a total reaction volume of 25 μl.

### Reference culture based method

The detection of *Salmonella *spp. was conducted in accordance with the recommendations from the Nordic Committee on Food Analyses (NMKL) [3] as previously described [13]. However, 25 g of sample (meat) or one swab was transferred to pre-heated buffered peptone water (1:10, BPW; Oxoid, Basingstoke, United Kingdom) and incubated at 37°C for 18 ± 2 h.

### Preparation of inoculum

To prepare the culture used for artificial inoculation in the comparative and collaborative trials, the *Salmonella *strains were grown overnight at 37°C on 5% blood agar (BA) plates (Statens Serum Institute, Copenhagen, Denmark). One colony of each of the strains was transferred to 4 ml of Nutrient broth with NaCl (8.5 g/l NaCl and 20 g/l Nutrient Broth (BD 234000, BD Denmark, Brøndby, Denmark)), vortexed and incubated at 37°C for 3–4 hours. After the incubation, a 10-fold dilution series in 0.9% NaCl solution was performed to determine the concentration of the *Salmonella *cells. From the dilution series, 0.1 ml from each tube was spread on two 5% BA plates. The tubes were stored at 2–5°C for 16 to 20 hours and the 5% BA plates were incubated for 16 to 20 hours at 37°C and the colonies were counted. The samples were subsequently inoculated from a tube in the dilution series with a known concentration of *Salmonella *cells. At the time of inoculation, 0.1 ml was spread onto each of two BA plates to estimate the actual inoculation level.

For the on-site validation, three different strains of *Salmonella *(two *S*. Infantis and one *S*. Agona) previously isolated from pork meat were grown in Brain Heart Infusion (Oxoid CM0225) at 37°C for 24 hours resulting in approximately 2 × 10^9 ^CFU/ml. The next day, the cultures were 10-fold diluted using 0.85% NaCl + 1% peptone.

### Sample preparation

Minced veal and pork meat were purchased at local retailers. Pig carcass swabs and poultry neck-skins were obtained from local abattoirs. Carcass swabs were sampled according to ISO 17604 [25] in accordance with EU directive 2073/2005/EC [26] employing the non-destructive swab method with gauze swabs. The sites on the pig carcass that were swabbed included the ham, back, belly and jowl.

After being transported cooled to the laboratory, the samples were analyzed using the real-time PCR method (DNA extraction and TaqMan PCR, as described above) and the reference culture method. Briefly, *Salmonella*-free (verified by the NMKL-71 method) fresh meat (25 g) or swab sample (one swab) was transferred to 225 ml (for meat samples) or 1:10 (weight of sample:volume of buffer for swabs) of BPW (37°C). Different levels of *Salmonella *(see "Comparative trial" and "Collaborative trial" below) were thereafter added. All the samples were pre-heated to 37°C and homogenized by hand for 20 seconds. After pre-enrichment at 37°C (12 ± 2 h for minced meat and neck-skins and 14 ± 1.5 for swabs), 5 ml aliquots were drawn for DNA-extraction and real-time PCR analysis using 9 μl of the extracted DNA. The enrichment was thereafter continued up to 18 hours according to NMKL-71 [3] and further analyzed according to that protocol.

### Comparative trial

The comparative trial was designed and conducted according to the recommendations from NordVal [15]. To evaluate the relative detection level, artificially inoculated samples were analyzed by NMKL-71 and the real-time PCR method as described above. For each of the matrices of minced meat, poultry neck-skins and pig carcass swabs, one sample of 25 g (for meat and neck-skins) and one swab was left un-inoculated; six were inoculated with 1–10 CFU/25 g and six with 10–100 CFU/25 g. Half of the samples at each inoculation level were inoculated with *S*. Enteritidis CCUG 32352 and the other half with *S*. Typhimurium CCUG 31969.

To evaluate the relative accuracy, relative specificity and relative sensitivity of the real-time PCR method, minced pork and veal meat (n = 60, artificially contaminated), poultry neck-skins (n = 60, artificially contaminated) and swabs from pig carcasses (n = 120, potentially naturally contaminated) were used, see Table 1. The samples were analyzed by NMKL-71 and the PCR method as described above. For the minced meat, 30 samples were left un-inoculated; 15 samples were inoculated with *S*. Livingstone (in-house bacteria culture collection) 1–10 CFU per 25 g and 15 samples were inoculated with *S*. Typhimurium CCUG 31969 1–10 CFU per 25 g. For the poultry neck-skins, 31 samples were left un-inoculated, 15 samples were inoculated with 1–10 CFU *S*. Enteritidis CCUG 32352 per 25 g and 14 samples were inoculated with 1–10 CFU *S*. Typhimurium CCUG 31969 per 25 g. The pig carcass swab samples consisted of 120 non-inoculated samples from a Danish abattoir.

### Collaborative trial

A collaborative trial involving six laboratories was performed to evaluate the robustness and reproducibility of the real-time PCR method testing identical samples. Laboratories belonging to Danish meat producers as well as other laboratories with the equipment used were selected for inclusion in the study. The reason for not including a larger number of participants was that it was not possible to find more than six laboratories that had the equipment and were willing to take part. The collaborative trial was designed and conducted according to the recommendations from NordVal [15] and included minced meat, poultry neck-skins and pig carcass swabs. The participating laboratories received pellets from 18 coded 5-ml samples (six from each matrix, see Table 2).

The samples for the collaborative trial were prepared as described above ("Sample preparation"). To produce the pellets included in the shipment, the supernatant was discarded after the centrifugation step, and the pellet kept at -20°C until shipped on ice to the trial participants. The samples were duplicate samples un-inoculated and inoculated artificially contaminated in duplicate with *S*. Typhimurium CCUG 31369 at two levels (1–10 CFU/25 g and 10–100 CFU/25 g) before enrichment, making it possible to assess the usefulness of the method at various infection levels.

The *Salmonella *status of all samples was confirmed by the reference culture method NMKL-71 [3] prior to and after spiking. The stability of the samples was examined using the real-time PCR method immediately after preparation, prior to commencement of the collaborative trial, during the period of analysis, as well after the trial was finished to verify the continued detection of *Salmonella*. The possible detrimental effect of shipping time at ambient temperature on the real-time PCR results was investigated, by analyzing a ring trial package after storage at room temperature for three days (the maximum shipment time to the participants was two days).

The shipment included a positive DNA control (1 μg/ml *S*. Typhimurium CCUG 31369) and a negative DNA control (1 μg/ml *Escherichia coli *O157 (Sample ID 077, Institute for Reference Materials and Measurements, Geel, Belgium)), a ready-to-use PCR mixture with added IAC, reagents for the magnetically based DNA extraction and the consumables for the DNA extraction and PCR analysis. To minimize any inter-laboratory variability (not attributable to the method performance), all the reagents necessary were supplied by the expert laboratory. At the participating laboratories, DNA extraction and PCR analysis were performed as described above. Real-time PCR at the participating laboratories was performed on an Mx3000 or Mx4000 real-time PCR system (Stratagene, La Jolla, CA). Each participant received a detailed protocol describing the DNA extraction, real-time PCR setup, real-time PCR run, and data analysis as well as a reporting form to record the obtained PCR results to return to the expert laboratory. The participants were also asked to return a file containing the real-time PCR runs. The participating laboratories were asked to use the negative template control (NTC), the process blank (a *Salmonella*-negative sample processed throughout the entire protocol) and the negative control to assign the threshold.

### External validation

Slices of pork filet were obtained from a local supermarket, and aseptically cut into pieces of 25 grams. Thirty-nine pieces of pork filet were inoculated by adding 0.5 ml of an appropriate dilution of *Salmonella *cells (see "Preparation of inoculum") onto the surface of the meat resulting in the following estimated inoculation levels for each of the three strains: one sample containing approximately 1000 CFU/25 g, one sample containing approximately 100 CFU/25 g, three samples containing approximately 10 CFU/25 g, four samples containing approximately 5 CFU/25 g and four samples containing approximately 2 CFU/25 g. After inoculation, the meat samples were placed in a stomacher bag and frozen at -18°C for 24 hours in order to induce a slight freezing stress to the *Salmonella*, resembling the stress during blast-cooling as used by the Danish abattoir.

All 39 samples were analyzed by the real-time PCR method and the BAX Salmonella Detection System (BAX, DuPont Qualicon, Oxoid) using the following protocol. The 25-g sample was thawed overnight at 4°C, 225 ml pre-warmed BPW (37°C, Oxoid) was added, and the samples were then incubated at 37°C. After 10 hours, a 5-ml aliquot was drawn for DNA extraction and subsequent real-time PCR analysis as described above. The remaining BPW was further incubated at 37°C for an additional 8 hours, and samples were thereafter treated according to the manufacturer's instructions.

### Statistical data analysis

The comparative validation study included three test characteristics: relative accuracy (AC), sensitivity (SE), and specificity (SP) (1) (see Table 1), and these were calculated and defined as previously described [27]. False negative (FN) results were defined as samples giving a negative result with PCR and a positive result with the NMKL-71 method. True positive (TP) results were defined as samples with positive PCR results and negative NMKL-71 results when obtained for artificially contaminated samples. Cohen's kappa (κ) was calculated as described by NMKL to quantify the degree of agreement between the two methods [28] (κ > 0.80 means very good agreement between the methods). This method was also used to evaluate the agreement between the real-time PCR and the BAX method in the on-site validation study.

For the collaborative validation study, the test reports and the real-time PCR analyses from the participating laboratories were carefully evaluated on return to the expert laboratory, and the results were approved for inclusion in the statistical analysis, unless they fell into at least one of the following two categories: (i) obvious performance deviation from the protocol and (ii) failed PCR analysis as shown in the included controls. The results obtained in the collaborative trial were analyzed according to the recommendations from NordVal [15]. SP was calculated for the un-inoculated samples by the following equation: SP = (1 - [FP/N-]) × 100%, where N- refers to the total number of samples not inoculated with Salmonella. SE was calculated for each level of spiking by the following equation: SE = (TP/N+) × 100%, where N+ refers to the number of artificially contaminated samples. AC was calculated for all levels of spiking by the following equation: AC = ([PA + NA + FP]/N) × 100%, where N refers to the number of samples tested.

## Authors' contributions

CL participated in the design of the study, performed part of the experimental work for the collaborative study, performed the statistical analysis and drafted the manuscript. MHJ and MK planned and performed the experimental work on the comparative study. FH planned and performed the experimental work for the external validation. JH conceived the study, obtained funding, helped to draft and critically read the manuscript. All authors read and approved the final manuscript.
